# Nasopharyngeal carriage, serotype distribution and antimicrobial resistance of *Streptococcus pneumoniae* among children from Brazil before the introduction of the 10-valent conjugate vaccine

**DOI:** 10.1186/1471-2334-13-318

**Published:** 2013-07-13

**Authors:** Felipe Piedade Gonçalves Neves, Tatiana Castro Abreu Pinto, Mariane Alves Corrêa, Roberta dos Anjos Barreto, Laís de Souza Gouveia Moreira, Havana Gomes Rodrigues, Claudete Araújo Cardoso, Rosana Rocha Barros, Lúcia Martins Teixeira

**Affiliations:** 1 Instituto Biomédico, Universidade Federal Fluminense, Niterói, RJ, Brazil; 2 Instituto de Microbiologia, Universidade Federal do Rio de Janeiro, Rio de Janeiro, RJ, Brazil; 3 Hospital Universitário Antônio Pedro, Universidade Federal Fluminense, Niterói, RJ, Brazil

**Keywords:** *Streptococcus pneumoniae*, Nasopharyngeal carriage, Serotypes, Antimicrobial resistance, Pneumococcal conjugate vaccines

## Abstract

**Background:**

*Streptococcus pneumoniae* remains a major cause of childhood morbidity and mortality worldwide. Nasopharyngeal colonization plays an important role in the development and transmission of pneumococcal diseases, and infants and young children are considered to be the main reservoir of this pathogen. The aim of this study was to evaluate the rates and characteristics associated with nasopharyngeal carriage, the distribution of serotypes and the antimicrobial resistance profiles of *Streptococcus pneumoniae* among children in a large metropolitan area in Brazil before the introduction of the 10-valent pneumococcal conjugate vaccine.

**Methods:**

Between March and June 2010, nasopharyngeal swabs were collected from 242 children aged <6 years attending one day care center and the emergency room of a pediatric hospital. Pneumococcal isolates were identified by conventional methods and serotypes were determined by a sequential multiplex PCR assay and/or the Quellung reaction. The antimicrobial susceptibilities of the pneumococci were assessed by the disk diffusion method. MICs for erythromycin and penicillin were also performed. Erythromycin resistance genes were investigated by PCR.

**Results:**

The overall colonization rate was 49.2% and it was considerably higher among children in the day care center. Pneumococcal carriage was more common among day care attenders and cohabitants with young siblings. The most prevalent serotypes were 6B, 19F, 6A, 14, 15C and 23F, which accounted for 61.2% of the isolates. All isolates were susceptible to clindamycin, levofloxacin, rifampicin and vancomycin. The highest rate of non-susceptibility was observed for sulphamethoxazole-trimethoprim (51.2%). Penicillin non-susceptible pneumococci (PNSP) accounted for 27.3% of the isolates (MICs of 0.12-4 μg/ml). Penicillin non-susceptibility was strongly associated with serotypes 14 and 23F. Hospital attendance and the presence of respiratory or general symptoms were frequently associated with PNSP carriage. The two erythromycin-resistant isolates (MICs of 2 and 4 μg/ml) belonged to serotype 6A, presented the M phenotype and harbored the *mef*(A/E) gene.

**Conclusions:**

Correlations between serotypes, settings and penicillin non-susceptibility were observed. Serotypes coverage projected for the 10-valent pneumococcal conjugate vaccine was low (45.5%), but pointed out the potential reduction of PNSP nasopharyngeal colonization by nearly 20%.

## Background

*Streptococcus pneumoniae* remains a major cause of childhood morbidity and mortality worldwide, particularly in lower income countries. Pneumococcal diseases are the leading source of vaccine preventable deaths, mostly due to community-acquired pneumonia (CAP) [1], accounting for approximately 11% of all deaths in children under 5 years old, excluding neonatal and/or HIV-positive deaths [2]. This microorganism is also frequently associated with bacteremia and meningitis, and it is the most common agent of acute otitis media (AOM) in young children [3].

Brazil was included among the fifteen countries with the highest estimated number of new cases of pneumonia [4]. Between 2000 and 2008, the mean annual rate of hospitalization due to CAP was 2,100/100,000 inhabitants: most cases (45%) occurred in children aged less than 5 years and were caused by *S. pneumoniae*[5]. The average incidence of pneumococcal meningitis during the 2000–2010 period was 1,227 cases/year, with a mean mortality rate around 30% [6]. In 2010, a survey on the occurrence of invasive pneumococcal diseases (IPD) in several Brazilian locations revealed that the majority of cases corresponded to meningitis (65.1%), followed by pneumonia (20.5%) and bacteremia (11.2%) [7]. In the single study available on the etiology of AOM among Brazilian patients, *S. pneumoniae* was reported as the prevalent agent [8], which is consistent with findings of other geographical regions [3].

More than 90 distinct pneumococcal serotypes have been described, based on chemical and antigenic variations of the capsular polysaccharide structure. The capsule is a major epidemiological marker for tracing pneumococcal infections, and constitutes the base of vaccine formulations currently available against this microorganism. At the present, two pneumococcal conjugate vaccines, the 10-valent (PCV10) and 13-valent (PCV13) vaccines are widely available, since the 7-valent (PCV7) is being progressively removed from the market [9].

PCV7 was introduced in Brazil in 2001, but it was available to a small portion of the population, since it was almost restricted to private clinics, and to individuals considered to be at high risk for IPD, who received vaccination by governmental agencies. Nevertheless, in March 2010, the PCV10 was included in the Brazilian Immunization Program, being offered as a free of charge universal childhood vaccination [5] and it has been, since then, gradually introduced in different areas of the country.

Nasopharyngeal colonization was found to play an important role in the development and transmission of pneumococcal diseases, and infants and young children are considered to be the main reservoir of this pathogen [9]. Thus, surveillance of colonization is important for vaccination monitoring process and pre-PCV10 era data are important to predict and assess its impact [10]. The aim of this study was to determine the prevalence of *S. pneumoniae* carriage among children aged less than 6 years attending two institutions in a large metropolitan area in Brazil, before the introduction of the PCV10.

## Methods

### Population

From March to June 2010, 242 children under 6 years of age attending one day care center (DCC; n=102) and the emergency room of a pediatric hospital (PH; n=140) in Niterói city were enrolled in the present study. Niterói city is a large metropolitan area located in the state of Rio de Janeiro in the Southeastern region of Brazil. The subjects enrolled in the study did not receive any dose of the PCV10, since this vaccination was introduced in this city only in October 2010. Both institutions are public and deliver services to low income population. The age group selected for this study reflects the age range of the children attending national DCCs.

### Study design

A single nasopharyngeal (NP) swab was obtained from each child, and their accompanying adults answered to an interviewer-administered questionnaire including demographic characteristics, symptomatology, if any, at admission in the study (fever, coryza/sneezing, cough/expectoration, fatigue/breathlessness, hipoactivity), day care attendance, chronic or recurrent disease, antibiotic used in the previous three months, prior hospitalization, young (aged <6 years) siblings, and if the child had received at least one dose of the PCV7.

### Isolation and identification of strains

NP specimens were obtained by using a mini tip flocked with Nylon fiber swab (code 518CS01; Copan, Brescia, Italy). Swabs were placed in a tube with 1.0 ml of skim milk tryptone-glucose-glycerin (STGG) transport medium. All specimens were processed within 6 hours after collection. NP swabs were streaked onto tryptic soy agar (Difco Laboratories, Detroit, MI, USA) containing 5% sheep blood and 5.0 μg/ml gentamicin (Sigma Chemical Co., St. Louis, MO, USA) and incubated at 36±1°C in 5% CO_2_-enriched atmosphere. Suspect colonies were isolated and identified according to α-hemolysis, susceptibility to optochin and bile-solubility. If different colony types were observed in a single culture plate, each colony type was separately identified.

### Determination of capsular serotypes

Serogroups or serotypes of pneumococcal isolates were deduced by a sequential multiplex PCR assay designed for Latin America [11,12]. To differentiate among *Streptococcus pneumoniae* serotypes 6A, 6B, and 6C, a previously described PCR protocol was employed [13]. Isolates not resolved by PCR were serotyped by the Quellung reaction with antisera kindly provided by the Centers for Disease Control and Prevention (CDC). Non-typeable (NT) isolates by both methods were also tested for specific amplification of the *lytA* gene [14].

### Antimicrobial susceptibility testing

The bacterial isolates were tested by the disk diffusion method according to the Clinical and Laboratory Standards Institute recommendations [15]. The antimicrobial agents tested were: chloramphenicol (30 μg), clindamycin (2 μg), erythromycin (15 μg), levofloxacin (5 μg), oxacillin (1 μg), rifampicin (5 μg), sulphamethoxazole-trimethoprim (23.75 μg / 1.25 μg), tetracycline (30 μg) and vancomycin (30 μg) (Cecon, São Paulo, SP, Brazil). Isolates with reduced susceptibility to penicillin (< 20 mm zone diameter around the oxacillin disk) and/or erythromycin (< 21 mm zone diameter) were submitted to determination of Minimum Inhibitory Concentrations (MIC) by using the M.I.C.Evaluator™ strips (Oxoid, Basingstoke, Hants, UK). The presence of macrolide resistance phenotypes was investigated by the double disk test using erythromycin (15 μg) and clindamycin (2 μg) disks apart 12 mm from each other [15].

### Detection of erythromycin resistance associated genes

The presence of the *erm*(A), *erm*(B) and *mef*(A/E) genes was evaluated among erythromycin non-susceptible isolates by PCR as previously described [16].

### Ethical considerations

The protocols for collection and informed consent were approved by the Ethics Committee of the Universidade Federal Fluminense (CAAE no. 0142.0.258.000-09). Written informed consent was obtained from parents or legal guardians.

## Results

Two hundred forty-two subjects aged less than 6 years attending two public institutions between March and June 2010 were included in this study. Their median age was 3 years old (interquartile range: 1.6 – 4.5 years old), 129 of them (53.3%) were male and 113 (46.7%) were female.

A total of 121 pneumococcal isolates was recovered from 119 children, revealing an overall colonization rate of 49.2%. Two phenotypically distinct pneumococcal colonies were detected in the respective cultures obtained from two subjects, indicating co-colonization. The prevalence of pneumococcal carriage was 42.1% (59/140) among children at the PH and 58.8% (60/102) among those at the DCC.

The demographical and clinical characteristics of the 242 children included in this study are shown in Table 1. The characteristics that showed greater differences between carriers and non-carriers were day care attendance and cohabiting with siblings under 6 years of age.

**Table 1:** Demographic and clinical characteristics of the 242 children enrolled in the present study

**Risk factor**	***S. pneumoniae *carriage**	
	**Yes (n=119)**	**No (n=123)**
Sex		
Male	61	68
Female	58	55
Age		
< 2 years	31	44
≥ 2 years	85	76
Symptomatology at admission in the study		
Yes	87	92
No	29	28
Fever		
Yes	45	42
No	71	78
Coryza/sneezing		
Yes	63	60
No	53	60
Cough/expectoration		
Yes	66	63
No	50	57
Fatigue/breathlessness		
Yes	24	29
No	92	91
Hipoactivity		
Yes	20	26
No	96	94
Chronic or recurrent disease		
Yes	27	26
No	89	94
Antibiotics in previous 3 months		
Yes	35	44
No	80	76
Prior hospitalization		
Yes	38	36
No	77	84
Day care attendance^a^		
Yes	92	71
No	27	52
Young (< 6 years) siblings		
Yes	61	40
No	55	80
Received at least 1 dose of PCV7		
Yes	2	4
No	113	116

^a^Day care attendance includes all children of the day care center plus those from hospital whose parents or legal guardians answered “Yes” to question “Does the child attend a day care center?”; PCV7, 7-valent pneumococcal conjugate vaccine; Data from seven children were not fully available.

Twenty-five serotypes were detected among 117 (96.7%) of the 121 pneumococcal isolates recovered. The *cpsA* pneumococcal identification control gene was not detected among the four non-typeable (NT) isolates, but their identification was confirmed by amplification of the *lytA* gene. The prevalent serotypes identified were 6B (15.7%), 19F (12.4%), 6A (9.9%), 14 (9.1%), 15C (8.3%) and 23F (5.8%). Table 2 shows the distribution of serotypes according to the institution.

**Table 2:** **Distribution of capsular types among 121 *Streptococcus pneumoniae *isolates recovered from the nasopharynx of children living in a large metropolitan area in Southeastern Brazil**

**Day care center (n=61)**		**Pediatric hospital (n=60)**	
**Serotypes**	**No. (%)**	**Serotypes**	**No. (%)**
6B	14 (23.0)	19F	13 (21.7)
15C	8 (13.1)	6A	8 (13.3)
17F	6 (9.8)	14	7 (11.7)
6A	4 (6.6)	23F	7 (11.7)
11A	4 (6.6)	6B	5 (8.3)
14	4 (6.6)	16F	4 (6.7)
6C	3 (4.9)	10A	2 (3.3)
19A	3 (4.9)	15C	2 (3.3)
23B	3 (4.9)	23A	2 (3.3)
15A	2 (3.3)	Others^b^	9 (15.0)
19F	2 (3.3)	NT	1 (1.7)
Others^a^	5 (8.2)		
NT	3 (4.9)		

^a^serotypes 3, 18B, 23A, 29 and 34.

^b^serotypes 4, 9N, 6C, 12F, 18C, 19A, 34, 35B and 39.

*NT* non-typeable.

Serotype 19F (21.7%; 13/60) was the prevalent type among carriers from the PH and the presence of respiratory symptoms such as coryza/sneezing and cough/expectoration showed to be associated with colonization by pneumococci belonging to this serotype. On the other hand, serotype 6B (23%; 14/61) was the most frequent among children attending the DCC, mainly in children aged ≥ 2 years. Regarding simultaneous colonization, in both the subjects, one of the isolates was NT and the remaining isolates belonged to serotypes 6B or 19A. Additionally, the serotypes recovered from the two pneumococcal carriers who had received only the first primary immunization dose of the PCV7, both attending the DCC, were 6B and 15C.

All the isolates were susceptible to clindamycin, levofloxacin, rifampicin and vancomycin. Susceptibility to the other five antimicrobial agents tested varied, with the highest percentage of non-susceptibility being observed for sulphamethoxazole-trimethoprim (51.2%; 62/121), including 38.8% resistant and 12.4% intermediate isolates.

**Figure 1:**
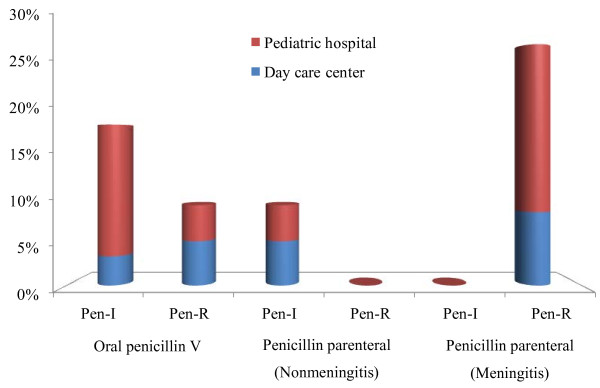
**Distribution of penicillin non-susceptible (MIC > 0.6 μg/ml) *Streptococcus pneumoniae *considering the different MIC breakpoints.** Pen, penicillin; R, resistant; I, intermediate. MIC, Minimum Inhibitory Concentrations. Adopted MIC breakpoints: oral penicillin V (I = 0.12-1.0 μg/ml; R ≥ 2.0 μg/ml); penicillin parenteral (nonmeningitis - I = 4.0 μg/ml; R ≥ 8.0 μg/ml); and penicillin parenteral (meningitis - R ≥ 0.12 μg/ml).

Non-susceptibility to penicillin was detected in 27.3% (33/121) of the isolates and MICs ranged from 0.12 to 4.0 μg/ml. Following the current CLSI criteria for oral penicillin V, 22 (18.2%) isolates presented intermediate MICs (Pen-I) and 11 (9.1%) showed MICs indicative of resistance to penicillin (Pen-R). According to the CLSI breakpoints for parenteral penicillin (meningitis), all 33 isolates would be considered Pen-R. However, for parenteral penicillin (nonmeningitis), only 11 (9.1%) isolates would be Pen-I and none would be Pen-R (Figure 1).

Penicillin non-susceptible pneumococci (PNSP) were more common among children attending the PH, being this characteristic strongly associated with serotypes 14 and 23F (Table 3). Nine (81.8%) of the 11 serotype 14 isolates were PNSP and all of them presented the highest penicillin MIC observed in this study (4.0 μg/ml). Likewise, all seven serotype 23F isolates were non-susceptible to penicillin, with MICs ranging from 0.18 μg/ml to 0.25 μg/ml. In addition, all the symptoms investigated in this study were strongly associated with PNSP carriage, including fever, coryza/sneezing, cough/expectoration, fatigue/breathlessness and hipoactivity.

**Table 3:** **Characteristics of penicillin non-susceptible (MIC > 0.6 μg/ml) *Streptococcus pneumoniae *serotypes recovered from nasopharyngeal carriers in a large metropolitan area in Southeastern Brazil**

**Penicillin MIC**	**Number of isolates (%)**	**Serotypes (number of isolates)**	**Institution (number of isolates)**
0.12 μg/ml	5 (15.1)	19F (3), 6B, 34	Pediatric hospital
0.18 μg/ml	2 (6.1)	19F, 23F	Pediatric hospital
0.375 μg/ml	2 (6.1)	6A, 23F	Pediatric hospital
0.25 μg/ml	11(33.3)	23F (5), 6B (3), NT (2), 10A	Pediatric hospital (7)
			Day care center (4)
0.5 μg/ml	2 (6.1)	6A (2)	Pediatric hospital
4.0 μg/ml	11 (33.3)	14 (9), NT (2)	Pediatric hospital (5)
			Day care center (6)
Total	33 (100)		

*MIC* Minimum Inhibitory Concentrations.

*NT* non-typeable.

Two isolates were non-susceptible to erythromycin, showing MICs of 2.0 μg/ml and 4.0 μg/ml. Both isolates were obtained from children at the PH (each one from a different child), belonged to serotype 6A, presented the M phenotype and harbored the *mef*(A/E) gene. Both isolates were also PNSP, with MICs of 0.25 μg/ml.

Resistance to tetracycline was observed in only two (1.7%) pneumococcal isolates (one serotype 6B and another NT) that colonized simultaneously the same child attending the DCC. Eight (6.6%) additional isolates were intermediate to tetracycline.

Four (3.3%) isolates were resistant to chloramphenicol, and two of them belonged to serotype 6B. The other two were NT and represented the only multiresistant isolates detected in this study, since they were also resistant to sulphamethoxazole-trimethoprim and non-susceptible to penicillin (MICs of 0.25 μg/ml).

Among the 140 children attending the PH that were included in the present study, seven (5.0%) were hospitalized with pneumonia, and four of them were also nasopharyngeal pneumococcal carriers. The isolates recovered from these individuals belonged to serotypes 19F (n=3) and 14 (n=1). All of them, except one serotype 19F isolate, were PNSP. However, no microorganisms were recovered from the blood culture of these patients, and after treatment using β-lactams the children had their health restored.

## Discussion

The frequency of *S. pneumoniae* NP carriage was investigated among children attending two distinct settings in a large metropolitan area in Brazil. An overall prevalence of 49.2% was found, and it was considerably higher at the DCC when compared to the PH (58.8% vs. 42.1%). The most common characteristics associated with pneumococcal colonization observed in this study (day care attendance and cohabitating with young siblings) were also found in recently published studies carried out in Taiwan [17] and in the USA [18], reinforcing our findings. Other factors might have accounted for the high prevalence rate in the present study, such as the low socioeconomic status of the analyzed population and crowding [19].

Data from the SIREVA II project (2010) revealed that the most prevalent serotypes associated with IPD among children less than 5 years old in Latin America were 14, 6B, 19A, 1, 23F, 6A and 19F, accounting for approximately 60% of these diseases [7]. Although we have not analyzed invasive isolates, these serotypes, except for serotype 1 which was not detected, represented 56.2% of the isolates recovered from the nasopharynx of children included in this study. Such observations highlight the importance of pneumococcal carriage, since frequency of isolation was very similar to the one observed for IPD, and previous colonization is known to be a step usually required for the development of invasive infections.

Considering that the currently available conjugate vaccines against *S. pneumoniae* may contribute to eliminate the asymptomatic carriage, PCV7 and PCV10 would have an identical theoretical impact (45.5%) in the analyzed population, since the additional serotypes represented in the PCV10 (1, 5 and 7F) were not detected. Regarding PCV13, which contains serotypes 3, 6A and 19A in addition to those provided in PCV10 and is also approved for use in Brazil, the projected coverage for pneumococci recovered in the present study was 59.5%. Nevertheless, in Brazil, this vaccine is currently available only in private clinics. By setting, this impact would be considerably lower among children attending the DCC when compared to those at the PH, as for PCV10 (34.4% vs. 56.7%) and for PCV13 (47.5% vs. 71.7%).

The reason behind this very low estimated coverage in the DCC is the prevalence of serotypes 15C, 17F and 11A, ranked from 2^nd^ to 4^th^ position respectively at this scenario, which are not included in any of the available conjugate vaccine formulations. These serotypes have also been found in the nasopharynx of unvaccinated children aged less than 5 years in a previous Brazilian study, but they are rarely associated with invasive diseases in Latin America and other regions of the world [7,20]. Interestingly, serotype 15B/C, together with 6C and 19A, was one of the serotypes most commonly isolated from children aged <7 years in primary care practices in Massachusetts, USA, where serotype replacement post-PCV7 is considered essentially complete [18]. These serotypes are already circulating among the children included in the present study, mostly at the DCC where they account for almost 25% of the pneumococcal isolates, and this finding can predict serotype replacement post-PCV10 in our region.

We have also observed association between serotypes, settings and penicillin non-susceptibility, since serotypes 6B and 19F were strongly associated with children at the DCC and the PH, respectively, whereas penicillin non-susceptibility was more common among pneumococci isolated from children at the PH, being frequently related to serotypes 14 and 23F.

As previously demonstrated [19], colonization rates tend to be higher during respiratory tract infections. Although, we have not observed this fact globally in the present study, serotype 19F carriage was strongly associated with children who presented respiratory symptoms, such as coryza/sneezing and cough/expectoration. Also, it was possible to observe that PNSP colonization was very frequent among children presenting all the symptoms assessed, both general and respiratory.

Despite the strong association of PNSP with serotypes 14 and 23F, non-susceptibility to penicillin was also found among serotypes 6A, 6B, 10A, and 19F. These results are in accordance with data in the literature, showing that most PNSP isolates belong to serotypes 6A, 6B, 9V, 14, 15A, 19F, 19A, and 23F [7,21,22]. Although this association is well-documented, non-susceptibility to penicillin has already been found in other serotypes, such as 3, 6C, and 18C [7], and in the present study, serotype 10A.

In theory, vaccine coverage projected for isolates recovered in the present study could potentially reduce penicillin non-susceptibility rates from 27.3% to 7.4% or 5% with the use of PCV10 or PCV13, respectively. However, other authors have described no or low impact on PNSP carriage post-PCV7 immunization [23,24]. Also, in a recent study carried out in Korea after optional use of the PCV7, rates of penicillin non-susceptibility were found to increase significantly and were strongly associated with non-vaccine serotypes 6A and 19A [25].

High rates of non-susceptibility to sulphamethoxazole-trimethoprim have already been described among invasive and carriage isolates recovered from Brazilian children and adolescents, corroborating the findings of the present study [7,26,27].

The low rate of erythromycin resistance observed in the present study was similar to that obtained for isolates recovered from individuals under 15 years of age with pneumonia [27], but considerably lower to that (15.0%) reported in 2010 on the SIREVA II project among children with invasive infections aged < 5 years [7].

Despite high rates of non-susceptibility to penicillin and sulphamethoxazole-trimethoprim, the pneumococcal isolates recovered from nasopharynx of the children analyzed in this study showed a high degree of susceptibility for the majority of the antimicrobial agents tested and, as previously reported [19,20], this may also reflect the characteristics of the pneumococcal isolates associated with diseases in our region.

## Conclusions

Our findings indicate a high prevalence of pneumococcal carriage among the population analyzed and reinforce previous observations indicating that asymptomatic colonization by these microorganisms is common among children, particularly in closed communities and household contacts. Correlations between serotypes, settings and penicillin non-susceptibility were observed. Serotypes coverage projected for the 10-valent pneumococcal conjugate vaccine was low, but pointed out the potential reduction of PNSP nasopharyngeal colonization by nearly 20%.

Since data on isolates from nasopharyngeal colonization provides relevant information on the potential burden of pneumococcal diseases, antimicrobial resistance and their prevention, continued monitoring of prevalent serotypes and antimicrobial resistance is an important tool to estimate and evaluate the impact of the pneumococcal conjugate vaccines. This is essentially important now in Brazil, where PCV10 has been recently introduced for routine immunization, since the epidemiology of pneumococcal diseases varies in response to a selective pressure, which may probably be in progress already.

## Abbreviations

AOM: Acute otitis media; CAP: Community-acquired pneumonia; CLSI: Clinical and Laboratory Standards Institute; DCC: Day care center; IPD: Invasive pneumococcal diseases; MIC: Minimum inhibitory concentration; NP: Nasopharyngeal; NT: Non-typeable; PCV: Pneumococcal conjugate vaccines; PH: Pediatric hospital; PNSP: Penicillin non-susceptible pneumococci; STGG: Skim milk tryptone-glucose-glycerin.

## Competing interests

Authors declare that they have no competing interests.

## Authors’ contributions

LMT, FPGN, RRB and CAC contributed to the overall design of the study. FPGN, TCAP, MAC, RRB and CAC participated in field and clinical aspects of the study. FPGN performed major experimental analyses and drafted the manuscript. LMT and TCAP helped draft the manuscript. TCAP, MAC, RAB, LSGM, and HGR provided technical assistances. All authors read and approved the final manuscript.

## Pre-publication history

The pre-publication history for this paper can be accessed here:

http://www.biomedcentral.com/1471-2334/13/318/prepub
